# A cross‐sectional study of the impact of COVID‐19 pandemic on the physical activity of Jordanian population

**DOI:** 10.1002/hsr2.896

**Published:** 2022-10-28

**Authors:** Sajeda Awadi, Almu'atasim Khamees, Walaa Almdallal, Mai Alzoubi, Lana Talafha, Ahmad M. Alsheikh, Baha Aldin Faiyoumi, Raed M. Al‐Zoubi, Mazhar S. Al Zoubi

**Affiliations:** ^1^ Faculty of Medicine Yarmouk University Irbid Jordan; ^2^ King Hussein Cancer Center Amman Jordan; ^3^ Hamad Medical Corporation Doha Qatar; ^4^ Department of Surgery, Surgical Research Section Hamad Medical Corporation Doha Qatar; ^5^ Department of Biomedical Sciences, College of Health Sciences, QU‐Health Qatar University Doha Qatar; ^6^ Department of Chemistry Jordan University of Science and Technology Irbid Jordan

**Keywords:** COVID‐19, exercise, health, lifestyle, lockdown, physical activity

## Abstract

**Background:**

Since the declaration of the COVID‐19 pandemic on 11th March 2020, the public health recommendations have applied lockdowns and restrictions to limit the spread of the disease. These measures determined outdoor activities and access to many forms of exercise.

**Objective:**

The primary objective of this study was to examine the impact of the COVID‐19 pandemic on the physical activity (PA) of the Jordanian population.

**Methods:**

A cross‐sectional online survey was designed using Google Forms services and distributed on social media platforms during the first two weeks of November 2020 to evaluate the exercise activity changes during the COVID‐19 pandemic in the study population. In this research, we included those who perform any form of physical activity (n = 1103). The data collected were analyzed using the Statistical Package for the Social Sciences (SPSS) version 26 (IBM SPSS Corp, SPSS Statistics ver. 26, USA). The categorical variables were summarized as frequencies and proportions and were compared using the Chi‐square. For all analyses, P‐value less than 0.05 was considered statistically significant.

**Results:**

A total of 1103 out of 2,511 (43.9%) respondents who participated in the survey were physically exercising during the COVID‐19 pandemic. 41.8% were exercising at an irregular frequency, while 21.5% were exercising daily, 16.8% were exercising three to five times a week, and 19.9% were exercising once or twice a week. Moreover, 282 (25.6%) respondents started doing some form of exercise during the pandemic. Those significantly were less than 18‐year‐old, male gender, were single, were non‐smokers, and had a diploma or bachelor's degree in a health‐related major. These changes in the level of exercise have been attributed by 57.8% of respondents to the health aspects where they realized the importance of exercising in strengthening the immune system against diseases, including COVID‐19.

**Conclusion:**

The current study showed that lockdown, due to the COVID‐19 pandemic, had a positive impact on the healthy lifestyle of the Jordanian population which was attributed to their feeling of the importance of sports practice on the immune system and the availability of time for those activities. However, the younger and individuals were more aware of the importance of these practices which highlights the importance of considering other age groups in future studies of healthy behaviors.

## 1 INTRODUCTION

Physical exercise is defined according to the World Health Organization (WHO) as “any bodily movement produced by skeletal muscles that require energy expenditure.” Physical activity (PA) refers to all movement, including that during leisure time, while in transport to and from places, or as part of one's work. Both moderate‐ and vigorous‐intensity PA improve health,^1^ reduce the all‐over morbidity and mortality in the general population, and are associated with a lower burden of noncommunicable diseases (including cancer, heart diseases, type 2 diabetes mellitus, and obesity).^2^, ^3^

After the WHO declared the novel coronavirus infection (COVID‐19) pandemic (severe acute respiratory syndrome coronavirus‐2) a global health crisis in March 2020,^4^, ^5^ the public health recommendations and governmental measures have applied lockdowns, curfews, and wide restrictions. Although these might help in fighting against the spread of infection, such measures have been shown to impose negative effects on population health by limiting outdoor activity and access to many forms of exercise (e.g., stay‐at‐home policies, closed gyms, no group gatherings, increased social distancing, and remote work). These enforcements are known to be detrimental to the population's health in the long run^6^ as physical exercise is believed to be one of the most powerful lifestyle measures to cope with stressful events^7^, ^8^ such as the COVID‐19 pandemic.

In the majority of emerging studies, the restrictions have resulted in lower overall PA^9^, ^10^, ^11^, ^12^ and access to exercise despite the increased availability of PA classes and guidance on social media during the pandemic.^13^ On the other hand, some other people take advantage of their time during the quarantine and increase their level of exercise.^6^, ^14^, ^15^

The effect of the COVID‐19 pandemic on PA and other healthy lifestyle measures is still under review. Nonetheless, this effect needs to be taken into consideration when applying isolation regulations to people and the benefits must be weighed against the risks to provide a health policy that is in people's best interest.^11^ Given all of the above, the COVID‐19 pandemic is having a significant impact on multiple lifestyle interventions including people's PA, whether these effects are positive or negative. The main aim of this study is to examine and discuss how these parameters changed during the quarantine among a Jordanian sample.

## 2 METHODOLOGY

This cross‐sectional study was conducted after the IRB approval (No.: DSR/2022/58) at Yarmouk University and following the principles of the Declaration of Helsinki. The consent form was also gained from the participants. A multi‐section online questionnaire was designed through Google Forms and then distributed and collected during the first 2 weeks of November 2020 during which the team has gathered sufficient data from a satisfactory sample size. The questionnaire inquiries are attached in Appendix A. Various online modalities were utilized to ensure a colossal spread of the survey, including the official Yarmouk University students' email service and several social media platforms such as Facebook, WhatsApp, and Telegram. The survey targeted the Jordanian population from all walks of life, the total number of 2511 participants, with ages ranging from 15 to 72 years, who completed the questionnaire had a plethora of varying characteristics including but not limited to demographic features, Occupations, and level of education. All individuals partaking in the study had given consent to use the information they provide to fulfill the aims of this study. All respondents who have lived in Jordan since the beginning of the COVID‐19 pandemic can fill out survey questions. However, only 1103 (43.9%) of participants were doing any form of physical exercise during the COVID‐19 pandemic. In the aim of this study, we include those who perform any form of PA (*n* = 1103). The data collected were analyzed using the Statistical Package for the Social Sciences (SPSS) version 26 (IBM SPSS Corp; SPSS Statistics ver. 26). The categorical variables were summarized as frequencies and proportions and were compared using the *χ* ^2^. For all analyses, *p* value less than 0.05 was considered statistically significant.

## 3 RESULTS

### 3.1 Sociodemographic characteristics

The data were collected from all governorates in Jordan with an overall of 2511 respondents. Only, 1103 of the respondents met the inclusion criteria and performed any form of exercise during the pandemic, their demographic features are shown in Table 1. The majority of them were females (73.8%) and 66.0% of them were in the age group between 18 and 35 years old. However, 72 (6.5%) were children under the age of 18 years. The response rate was higher among those who live in a city; accounting for around 76.0% of the total responses. Seven hundred and six (64.0%) of them were single and 568 (51.5%) lived in a family of four to six members. Pointing to the respondent's level of education, 806  (73.1%) of them had a diploma or bachelor's degree including 357 (32.4%) who have a degree in a health‐related major while 68.9% of them were unemployed. Nine hundred eighty‐nine (89.7%) do not suffer from any chronic illnesses and 879 (81.3%) were nonsmokers.

**Table 1:** Demographic characteristics of the participants who perform physical activity (*n* = 1103)

Age	
Under 18 years	72 (6.5%)
18−35 years	728 (66.0%)
Above age 35 years	303 (27.5%)
Gender	
Male	289 (26.2%)
Female	814 (73.8%)
Social status	
Single	706 (64.0%)
Married	397 (36.0%)
Number of household members	
3 or less	202 (18.3%)
4−6	568 (51.5%)
7 or more	333 (30.2%)
Educational level	
High school diploma or still in school	157 (14.2%)
Diploma or bachelor's degree in a health‐related major	357 (32.4%)
Diploma or bachelor's degree in a non‐health‐related major	449 (40.7%)
Master's degree or PhD	140 (12.7%)
Occupation	
Health‐related field	88 (8.0%)
Nonhealth‐related field	255 (23.1%)
I don't work	760 (68.9%)
Place of residence	
City	838 (76.0%)
Village	252 (22.8%)
Refugee camp	13 (1.2%)
Do you suffer from any chronic illnesses?	
Yes	114 (10.3%)
No	989 (89.7%)
Do you currently smoke (this includes regular cigarettes, electronic cigarettes, hookah, etc.)?	
Yes	206 (18.7%)
No	897 (81.3%)

### 3.2 PA

**Figure 1:**
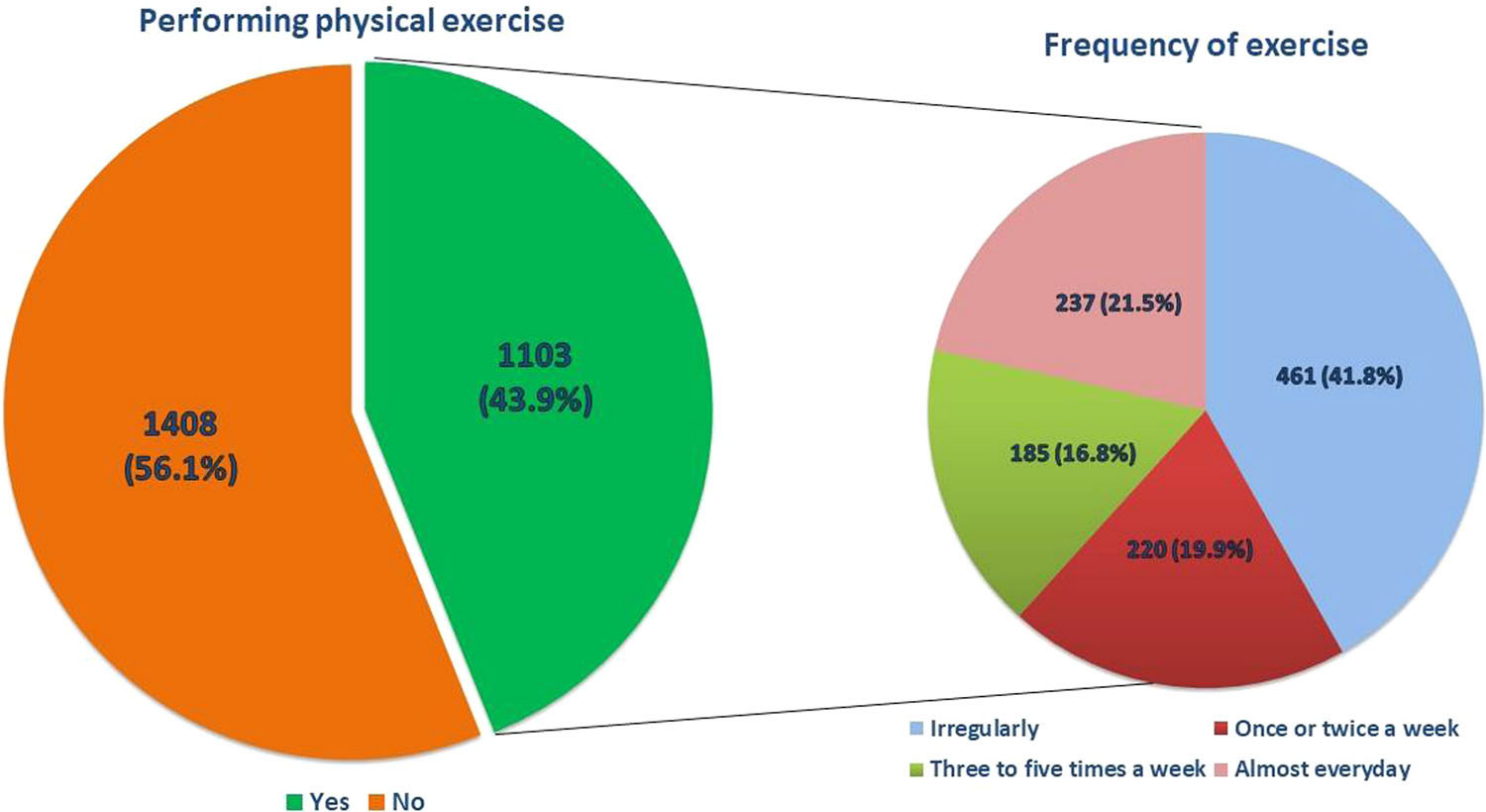
Respondents who perform any physical exercise since the start of the pandemic (*n* = 2511). The frequency of exercise for those who perform physical activity (*n* = 1103) (Irregularly, once or twice a week, three to five times a week, almost everyday).

**Figure 2:**
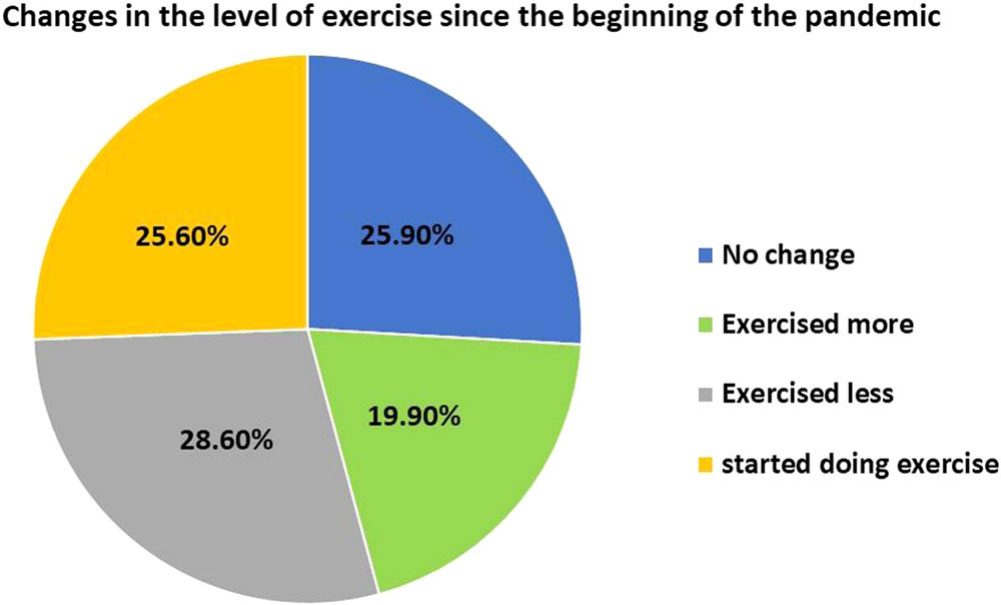
Changes in the level of exercise since the beginning of the pandemic (*n* = 1103). About 25.6% of respondents started doing exercise during the pandemic, 28.6% exercised less, and 19.9% exercised more. While the remaining (25.9%) had no changes in their level of exercise.

Nearly 1103 (43.9%) respondents were doing a minimum of one form of physical exercise during the COVID‐19 pandemic; 237 (21.5%) of them were exercising almost everyday, 185 (16.8%) were exercising three to five times a week, and 461 (41.8%) were exercising in an irregular frequency (Figure 1). In addition, individuals who were performing PA (*n* = 1103) showed that 316 (28.6%) have realized the importance of exercise but did not accordingly include it in their daily routine, so they exercised less since the beginning of the pandemic. While 282 (25.6%) have started doing some form of exercise, and 219 (19.9%) have regularly exercised before the pandemic and they have been more driven to exercise since the pandemic started (Figure 2). (Tables 2, 3, 4).

**Table 2:** Association between performing physical exercise (since the start of the pandemic) and the other variables (*n* = 2511)

		Do you currently perform any form of physical exercise (this includes walking, jogging, or any activity requiring physical exertion)?			*p* Value
		Yes	No	Total	
Age	Under 18 years	72 (2.9%)	79 (3.1%)	151 (6.0%)	0.617
	18−35 years	728 (29.0%)	933 (37.2%)	1661 (66.1%)	
	Over 35 years	303 (12.1%)	396 (15.8%)	699 (27.8%)	
Gender	Male	289 (11.5%)	281 (11.2%)	570 (22.7%)	**0.000**
	Female	814 (32.4%)	1127 (44.9%)	1941 (77.3%)	
Social status	Single	706 (28.1%)	853 (34.0%)	1559 (62.1%)	0.079
	Married	397 (15.8%)	555 (15.8%)	952 (37.9%)	
Number of household members	3 or less	202 (8.0%)	214 (8.5%)	416 (16.6%)	0.112
	4−6	568 (22.6%)	748 (29.8%)	1316 (52.4%)	
	7 or more	333 (13.3%)	446 (17.8%)	779 (31%)	
Educational level	High school diploma or still in school	157 (6.3%)	223 (8.9%)	380 (15.1%)	0.627
	Diploma or bachelor's degree in a health‐related major	357 (14.2%)	463 (18.4%)	820 (32.7%)	
	Diploma or bachelor's degree in non‐health‐related major	449 (17.9%)	556 (22.1%)	1005 (40.0%)	
	Master's degree or PhD	140 (5.6%)	166 (6.6%)	306 (12.2%)	
Occupation	Health‐related field	88 (3.5%)	108 (4.3%)	196 (7.8%)	0.850
	Nonhealth‐related field	255 (10.2%)	315 (12.5%)	570 (22.7%)	
	I don't work	760 (30.3%)	985 (39.2%)	1745 (69.5%)	
Place of residence	City	838 (33.4%)	1035 (41.2%)	1873 (74.6%)	0.125
	Village	252 (10.0%)	363 (14.5%)	615 (24.5%)	
	Refugee camp	13 (0.5%)	10 (0.4%)	23 (0.9%)	
Do you suffer from any chronic illnesses?	Yes	114 (4.5%)	166 (6.6%)	280 (11.2%)	0.251
	No	989 (39.4%)	1242 (49.5%)	2231 (88.8%)	
Fast food intake (after the start of the pandemic)	Once a month or less	697 (27.8%)	844 (33.6%)	1541 (61.4%)	0.139
	Once a week	272 (10.8%)	402 (16.0%)	674 (26.9%)	
	Three to four times a week	95 (3.8%)	107 (4.3%)	202 (8.0%)	
	Almost daily	39 (1.6%)	54 (2.2%)	93 (3.7%)	
Sugar intake (after the start of the pandemic)	Once a month or less	145 (5.8%)	107 (4.3%)	252 (10.0%)	**0.000**
	Once a week	321 (12.8%)	351 (14.0%)	672 (26.8%)	
	Three to four times a week	345 (13.7%)	444 (17.7%)	789 (31.4%)	
	Almost daily	292 (11.6%)	506 (20.2%)	798 (31.8%)	
Fat and oil intake (after the start of the pandemic)	Once a month or less	141 (5.6%)	101 (4.0%)	242 (9.6%)	**0.000**
	Once a week	279 (11.1%)	282 (11.2%)	561 (22.3%)	
	Three to four times a week	398 (15.9%)	513 (20.4%)	911 (36.3%)	
	Almost daily	285 (11.4%)	512 (20.4%)	797 (31.7%)	
Fresh fruit and vegetable intake (after the start of the pandemic)	Once a month or less	37 (1.5%)	55 (2.2%)	92 (3.7%)	**0.000**
	Once a week	77 (3.1%)	177 (7.1%)	254 (10.1%)	
	Three to four times a week	295 (11.8%)	424 (16.9%)	719 (28.6%)	
	Almost daily	694 (27.6%)	751 (29.9%)	1445 (57.6%)	
(Since the start of the pandemic) have you noticed any change in your weight?	I have gained weight	357 (14.2%)	556 (22.1%)	913 (36.4%)	**0.000**
	I have lost weight	250 (10.0%)	223 (8.9%)	473 (18.8%)	
	My weight hasn't changed	425 (16.9%)	536 (21.3%)	961 (38.3%)	
	I don't know	71 (2.8%)	93 (3.7%)	164 (6.5%)	
(Since the start of the pandemic) has the number of meals you eat in a day changed?	Increased	335 (13.3%)	509 (20.3%)	844 (33.6%)	**0.001**
	Decreased	208 (8.3%)	199 (7.9%)	407 (16.3%)	
	No change	560 (22.3%)	700 (27.9%)	1260 (50.2%)	
(Since the start of the pandemic) has the number of meals you eat during nighttime increased?	Yes	400 (15.9%)	605 (24.1%)	1005 (40.0%)	**0.001**
	No	703 (28.0%)	803 (32.0%)	1506 (60.0%)	
(Since the start of the pandemic) have you noticed any change in your appetite?	My appetite has increased	382 (15.2%)	563 (22.4%)	945 (37.6%)	**0.020**
	My appetite has decreased	194 (7.7%)	218 (8.7%)	412 (16.4%)	
	My appetite has not changed	527 (21.0%)	627 (25.0%)	1154 (46.0%)	
Downloading any application or started following any social media account concerning Healthy Nutrition	Yes	398 (15.9%)	320 (12.7%)	718 (28.6%)	**0.000**
	No	705 (28.1%)	1088 (43.3%)	1793 (71.4%)	
Downloading any application or started following any social media account concerning Physical Activity and Exercise	Yes	580 (23.1%)	346 (13.8%)	926 (36.9%)	**0.000**
	No	523 (20.8%)	1062 (42.3%)	1585 (63.1%)	
During the pandemic, how many hours do you sleep per day?	Less than 7 h	269 (10.7%)	303 (12.1%)	572 (22.8%)	**0.000**
	7−9 h	636 (25.3%)	752 (29.9%)	1388 (55.3%)	
	More than 9 h	198 (7.9%)	353 (14.1%)	551 (21.9%)	
Since the beginning of the pandemic, have you noticed a change in your sleep hours per day?	I started sleeping less	155 (6.2%)	184 (7.3%)	339 (13.5%)	0.250
	I started sleeping more	420 (16.7%)	582 (23.2%)	1002 (39.9%)	
	No change	528 (21.0%)	642 (25.6%)	1170 (46.6%)	
Do you currently smoke (this includes regular cigarettes, electronic cigarettes, hookah, etc.)?	Yes	206 (8.2%)	272 (10.8%)	478 (19.0%)	0.684
	No	897 (35.7%)	1136 (45.2%)	2033 (81.0%)	
Total		1103 (43.9%)	1408 (56.1%)	2511 (100%)	

*Note*: Values are expressed as the number and percentage of total respondents (*n* [%]). Variables are considered significant at *p* < 0.05 and marked in bold.

**Table 3:** Association between the frequency of exercise and the other variables (*n* = 1103)

		Frequency of exercise					*p* Value
		Irregularly	Once or twice a week	Three to five times a week	Almost everyday	Total	
Age	Under 18 years	40 (3.6%)	8 (0.7%)	8 (0.7%)	16 (1.5%)	72 (6.5%)	
	18−35 years	293 (26.6%)	154 (14.0%)	132 (12.0%)	149 (13.5%)	728 (66.0%)	0.086
	Over 35 years	128 (11.6%)	58 (5.3%)	45 (4.1%)	72 (6.5%)	303 (27.5%)	
Gender	Male	103 (9.3%)	47 (4.3%)	73 (6.6%)	66 (6.0%)	289 (26.2%)	**0.000**
	Female	358 (32.5%)	173 (15.7%)	112 (10.2%)	171 (15.5%)	814 (73.8%)	
Social status	Single	289 (26.2%)	140 (12.7%)	126 (11.4%)	151 (13.7%)	706 (64.0%)	0.633
	Married	172 (15.6%)	80 (7.3%)	59 (5.3%)	86 (7.8%)	397 (36.0%)	
Number of household members	3 or less	86 (7.8%)	51 (4.6%)	33 (3.0%)	32 (2.9%)	202 (18.3%)	
	4−6	218 (19.8%)	108 (9.8%)	104 (9.4%)	138 (12.5%)	568 (51.5%)	**0.022**
	7 or more	157 (14.2%)	61 (5.5%)	48 (4.4%)	67 (6.1%)	333 (30.2%)	
Educational level	High school diploma or still in school	71 (6.4%)	26 (2.4%)	19 (1.7%)	41 (3.7%)	157 (14.2%)	
	Diploma or bachelor's degree in a health‐related major	137 (12.4%)	82 (7.4%)	75 (6.8%)	63 (5.7%)	357 (32.4%)	0.068
	Diploma or bachelor's degree in non‐health‐related major	192 (17.4%)	83 (7.5%)	72 (6.5%)	102 (9.2%)	449 (40.7%)	
	Master's degree or PhD	61 (5.5%)	29 (2.6%)	19 (1.7%)	31 (2.8%)	140 12.7%)	
Occupation	Health‐related field	34 (3.1%)	22 (2.0%)	22 (2.0%)	10 (0.9%)	88 (8.0%)	
	Nonhealth‐related field	110 (10.0%)	48 (4.4%)	41 (3.7%)	56 (5.1%)	255 (23.1%)	0.122
	I don't work	317 (28.7%)	150 (13.6%)	122 (11.1%)	171 (15.5%)	760 (68.9%)	
Place of residence	City	352 (31.9%)	162 (14.7%)	158 (14.3%)	166 (15.0%)	838 (76.0%)	
	Village	106 (9.6%)	56 (5.1%)	25 (2.3%)	65 (5.9%)	252 (22.8%)	**0.006**
	Refugee camp	3 (0.3%)	2 (0.2%)	2 (0.2%)	6 (0.5%)	13 (1.2%)	
Do you suffer from any chronic illnesses?	Yes	43 (3.9%)	24 (2.2%)	17 (1.5%)	30 (2.7%)	114 (10.3%)	0.527
	No	418 (37.9%)	196 (17.8%)	168 (15.2%)	207 (18.8%)	989 (89.7%)	
Have you noticed any change in your level of exercise or even a change in the way you think of exercise since the beginning of the pandemic?	No change	144 (13.1%)	46 (4.2%)	35 (3.2%)	61 (5.5%)	286 (25.9%)	
	I have realized the importance of exercise but do not necessarily apply it to my daily routine	203 (18.4%)	60 (5.4%)	28 (2.5%)	25 (2.3%)	316 (28.6%)	**0.000**
	I have started doing some form of exercise.	92 (8.3%)	82 (7.4%)	58 (5.3%)	50 (4.5%)	282 (25.6%)	
	I regularly worked out before the pandemic but I am even more driven to exercise since the pandemic began	22 (2.0%)	32 (2.9%)	64 (5.8%)	101 (9.2%)	219 (19.9%)	
Fast food intake (after the start of the pandemic)	Once a month or less	299 (27.1%)	131 (11.9%)	109 (9.9%)	158 (14.3%)	697 (63.2%)	
	Once a week	106 (9.6%)	63 (5.7%)	50 (4.5%)	53 (4.8%)	272 (24.7%)	0.586
	Three to four times a week	37 (3.4%)	18 (1.6%)	21 (1.9%)	19 (1.7%)	95 (8.6%)	
	Almost daily	19 (1.7%)	8 (0.7%)	5 (0.5%)	7 (0.6%)	39 (3.5%)	
Sugar intake (after the start of the pandemic)	Once a month or less	47 (4.3%)	23 (2.1%)	34 (3.1%)	41 (3.7%)	145 (13.1%)	
	Once a week	131 (11.9%)	63 (5.7%)	62 (5.6%)	65 (5.9%)	321 (29.1%)	**0.014**
	Three to four times a week	147 (13.3%)	76 (6.9%)	55 (5.0%)	67 (6.1%)	345 (31.3%)	
	Almost daily	136 (12.3%)	58 (5.3%)	34 (3.1%)	64 (5.8%)	292 (26.5%)	
Fat and oil intake (after the start of the pandemic)	Once a month or less	40 (3.6%)	39 (3.5%)	25 (2.3%)	37 (3.4%)	141 (12.8%)	
	Once a week	111 (10.1%)	54 (4.9%)	53 (4.8%)	61 (5.5%)	279 (25.3%)	**0.027**
	Three to four times a week	174 (15.8%)	77 (7.0%)	68 (6.2%)	79 (7.2%)	398 (36.1%)	
	Almost daily	136 (12.3%)	50 (4.5%)	39 (3.5%)	60 (5.4%)	285 (25.8%)	
Fresh fruit and vegetable intake (after the start of the pandemic)	Once a month or less	22 (2.0%)	7 (0.6%)	2 (0.2%)	6 (0.5%)	37 (3.4%)	
	Once a week	38 (3.4%)	19 (1.7%)	10 (0.9%)	10 (0.9%)	77 (7.0%)	**0.017**
	Three to four times a week	121 (11.0%)	56 (5.1%)	64 (5.8%)	54 (4.9%)	295 (26.7%)	
	Almost daily	280 (25.4%)	138 (12.5%)	109 (9.9%)	167 (15.1%)	694 (62.9%)	
(Since the start of the pandemic) have you noticed any change in your weight?	I have gained weight	170 (15.4%)	66 (6.0%)	51 (4.6%)	70 (6.3%)	357 (32.4%)	
	I have lost weight	83 (7.5%)	61 (5.5%)	55 (5.0%)	51 (4.6%)	250 (22.7%)	**0.010**
	My weight hasn't changed	174 (15.8%)	81 (7.3%)	73 (6.6%)	97 (8.8%)	425 (38.5%)	
	I don't know	34 (3.1%)	12 (1.1%)	6 (0.5%)	19 (1.7%)	71 (6.4%)	
(Since the start of the pandemic) has the number of meals you eat in a day changed?	Increased	152 (13.8%)	68 (6.2%)	46 (4.2%)	69 (6.3%)	335 (30.4%)	
	Decreased	69 (6.3%)	50 (4.5%)	47 (4.3%)	42 (3.8%)	208 (18.9%)	**0.028**
	No change	240 (21.8%)	102 (9.2%)	92 (8.3%)	126 (11.4%)	560 (50.8%)	
(Since the start of the pandemic) has the number of meals you eat during nighttime increased?	Yes	180 (16.3%)	77 (7.0%)	63 (5.7%)	80 (7.3%)	400 (36.3%)	0.435
	No	281 (25.5%)	143 (13.0%)	122 (11.1%)	157 (14.2%)	703 (63.7%)	
(Since the start of the pandemic) have you noticed any change in your appetite?	Increased	169 (15.3%)	80 (7.3%)	53 (4.8%)	80 (7.3%)	382 (34.6%)	
	Decreased	66 (6.0%)	44 (4.0%)	39 (3.5%)	45 (4.1%)	194 (17.6%)	0.175
	No change	226 (20.5%)	96 (8.7%)	93 (8.4%)	112 (10.2%)	527 (47.8%)	
Downloading any application or started following any social media account concerning Healthy Nutrition	Yes	137 (12.4%)	93 (8.4%)	77 (7.0%)	91 (8.3%)	398 (36.1%)	**0.002**
	No	324 (29.4%)	127 (11.5%)	108 (9.8%)	146 (13.2%)	705 (63.9%)	
Downloading any application or started following any social media account concerning Physical Activity and Exercise	Yes	204 (18.5%)	132 (12.0%)	126 (11.4%)	118 (10.7%)	580 (52.6%)	**0.000**
	No	257 (23.3%)	88 (8.0%)	59 (5.3%)	119 (10.8%)	523 (47.4%)	
During the pandemic, how many hours do you sleep per day?	Less than 7 h	110 (10.0%)	46 (4.2%)	41 (3.7%)	72 (6.5%)	269 (24.4%)	
	7−9 h	269 (24.4%)	121 (11.0%)	121 (11.0%)	125 (11.3%)	636 (57.7%)	**0.013**
	More than 9 h	82 (7.4%)	53 (4.8%)	23 (2.1%)	40 (3.6%)	198 (18.0%)	
Since the beginning of the pandemic, have you noticed a change regarding your sleep hours per day?	Less	63 (5.7%)	24 (2.2%)	28 (2.5%)	40 (3.6%)	155 (14.1%)	
	More	177 (16.0%)	100 (9.1%)	67 (6.1%)	76 (6.9%)	420 (38.1%)	0.124
	No change	221 (20.0%)	96 (8.7%)	90 (8.2%)	121 (11.0%)	528 47.9%)	
Do you currently smoke (this includes regular cigarettes, electronic cigarettes, hookah, etc.)?	Yes	90 (8.2%)	30 (2.7%)	40 (3.6%)	46 (4.2%)	206 (18.7%)	0.169
	No	371 (33.6%)	190 (17.2%)	145 (13.1%)	191 (17.3%)	897 (81.3%)	
Total		461 (41.8%)	220 (19.9%)	185 (16.8%)	237 (21.5%)	1103 (100.0%)	

*Note*: Values are expressed as the number and percentage of total respondents (*n* [%]). Variables are considered significant at *p*< 0.05 and marked in bold.

**Table 4:** Association between changes in the level of exercise since the beginning of the pandemic and the other variables (*n* = 1103)

		Have you noticed any change in your level of exercise or even a change in the way you think of exercise since the beginning of the pandemic?					*p* Value
		No change	Exercised less	started doing some form of exercise	Exercised more	Total	
Age	Under 18 years	25 (2.3%)	10 (0.9%)	21 (1.9%)	16 (1.5%)	72 (6.5%)	
	18−35 years	185 (16.8%)	220 (19.9%)	201 (18.2%)	122 (11.1%)	728 (66.0%)	**0.000**
	Over 35 years	76 (6.9%)	86 (7.8%)	60 (5.4%)	81 (7.3%)	303 (27.5%)	
Gender	Male	81 (7.3%)	76 (6.9%)	56 (5.1%)	76 (6.9%)	289 (26.2%)	**0.001**
	Female	205 (18.6%)	240 (21.8%)	226 (20.5%)	143 (13.0%)	814 (73.8%)	
Social status	Single	185 (16.8%)	197 (17.9%)	204 (18.5%)	120 (10.9%)	706 (64.0%)	**0.001**
	Married	101 (9.2%)	119 (10.8%)	78 (7.1%)	99 (9.0%)	397 (36.0%)	
Number of household members	3 or less	51 (4.6%)	65 (5.9%)	49 (4.4%)	37 (3.4%)	202 (18.3%)	
	4−6	142 (12.9%)	153 (13.9%)	145 (13.1%)	128 (11.6%)	568 (51.5%)	0.319
	7 or more	93 (8.4%)	98 (8.9%)	88 (8.0%)	54 (4.9%)	333 (30.2%)	
Educational level	High school diploma or still in school	53 (4.8%)	34 (3.1%)	39 (3.5%)	31 (2.8%)	157 (14.2%)	
	Diploma or bachelor's degree in a health‐related major	80 (7.3%)	105 (9.5%)	111 (10.1%)	61 (5.5%)	357 (32.4%)	
	Diploma or bachelor's degree in nonhealth‐related major	111 (10.1%)	147 (13.3%)	102 (9.2%)	89 (8.1%)	449 (40.7%)	**0.002**
	Master's degree or PhD	42 (3.8%)	30 (2.7%)	30 (2.7%)	38 (3.4%)	140 (12.7%)	
Occupation	Health‐related field	25 (2.3%)	20 (1.8%)	24 (2.2%)	19 (1.7%)	88 (8.0%)	
	Nonhealth‐related field	66 (6.0%)	77 (7.0%)	57 (5.2%)	55 (5.0%)	255 (23.1%)	0.727
	I don't work	195 (17.7%)	219 (19.9%)	201 (18.2%)	145 (13.1%)	760 (68.9%)	
Place of residence	City	205 (18.6%)	245 (22.2%)	223 (20.2%)	165 (15.0%)	838 (76.0%)	0.399
	Village	77 (7.0%)	69 (6.3%)	56 (5.1%)	50 (4.5%)	252 (22.8%)	
	Refugee camp	4 (0.4%)	2 (0.2%)	3 (0.3%)	4 (0.4%)	13 (1.2%)	
Do you suffer from any chronic illnesses?	Yes	25 (2.3%)	29 (2.6%)	2 (2.4%)	33 (3.0%)	114 (10.3%)	0.082
	No	261 (23.7%)	287 (26.0%)	255 (23.1%)	186 (16.9%)	989 (89.7%)	
Frequency of exercise	Irregularly	144 (13.1%)	203 (18.4%)	92 (8.3%)	22 (2.0%)	461 (41.8%)	
	Once or twice a week	46 (4.2%)	60 (5.4%)	82 (7.4%)	32 (2.9%)	220 (19.9%)	
	Three to five times a week	35 (3.2%)	28 (2.5%)	58 (5.3%)	64 (5.8%)	185 (16.8%)	**0.000**
	Almost everyday	61 (5.5%)	25 (2.3%)	50 (4.5%)	101 (9.2%)	237 (21.5%)	
Fast food intake (after the start of the pandemic)	Once a month or less	189 (17.1%)	195 (17.7%)	165 (15.0%)	148 (13.4%)	697 (63.2%)	
	Once a week	64 (5.8%)	72 (6.5%)	86 (7.8%)	50 (4.5%)	272 (24.7%)	
	Three to four times a week	17 (1.5%)	40 (3.6%)	21 (1.9%)	17 (1.5%)	95 (8.6%)	**0.009**
	Almost daily	16 (1.5%)	9 (0.8%)	10 (0.9%)	4 (0.4%)	39 (3.5%)	
Sugar intake (after the start of the pandemic)	Once a month or less	37 (3.4%)	37 (3.4%)	28 (2.5%)	43 (3.9%)	145 (13.1%)	
	Once a week	86 (7.8%)	81 (7.3%)	78 (7.1%)	76 (6.9%)	321 (29.1%)	
	Three to four times a week	83 (7.5%)	102 (9.2%)	102 (9.2%)	58 (5.3%)	345 (31.3%)	**0.004**
	Almost daily	80 (7.3%)	96 (8.7%)	74 (6.7%)	42 (3.8%)	292 (26.5%)	
Fat and oil intake (after the start of the pandemic)	Once a month or less	36 (3.3%)	33 (3.0%)	30 (2.7%)	42 (3.8%)	141 (12.8%)	
	Once a week	58 (5.3%)	83 (7.5%)	77 (7.0%)	61 (5.5%)	279 (25.3%)	
	Three to four times a week	101 (9.2%)	114 (10.3%)	106 (9.6%)	77 (7.0%)	398 (36.1%)	**0.005**
	Almost daily	91 (8.3%)	86 (7.8%)	69 (6.3%)	39 (3.5%)	285 (25.8%)	
Fresh fruit and vegetable intake (after the start of the pandemic)	Once a month or less	16 (1.5%)	12 (1.1%)	5 (0.5%)	4 (0.4%)	37 (3.4%)	
	Once a week	19 (1.7%)	32 (2.9%)	18 (1.6%)	8 (0.7%)	77 (7.0%)	
	Three to four times a week	71 (6.4%)	87 (7.9%)	84 (7.6%)	53 (4.8%)	295 (26.7%)	**0.012**
	Almost daily	180 (16.3%)	185 (16.8%)	175 (15.9%)	154 (14.0%)	694 (62.9%)	
(Since the start of the pandemic) have you noticed any change in your weight?	I have gained weight	85 (7.7%)	133 (12.1%)	82 (7.4%)	57 (5.2%)	357 (32.4%)	
	I have lost weight	58 (5.3%)	53 (4.8%)	86 (7.8%)	53 (4.8%)	250 (22.7%)	
	My weight hasn't changed	117 (10.6%)	110 (10.0%)	98 (8.9%)	100 (9.1%)	425 (38.5%)	**0.000**
	I don't know	26 (2.4%)	20 (1.8%)	16 (1.5%)	9 (0.8%)	71 (6.4%)	
(Since the start of the pandemic) has the number of meals you eat in a day changed?	Increased	75 (6.8%)	115 (10.4%)	86 (7.8%)	59 (5.3%)	335 (30.4%)	
	Decreased	46 (4.2%)	51 (4.6%)	70 (6.3%)	41 (3.7%)	208 (18.9%)	**0.003**
	No change	165 (15.0%)	150 (13.6%)	126 (11.4%)	119 (10.8%)	560 (50.8%)	
(Since the start of the pandemic) has the number of meals you eat during nighttime increased?	Yes	92 (8.3%)	132 (12.0%)	107 (9.7%)	69 (6.3%)	400 (36.3%)	**0.033**
	No	194 (17.6%)	184 (16.7%)	175 (15.9%)	150 (13.6%)	703 (63.7%)	
(Since the start of the pandemic) have you noticed any change in your appetite?	Increased	85 (7.7%)	128 (11.6%)	97 (8.8%)	72 (6.5%)	382 (34.6%)	
	Decreased	43 (3.9%)	48 (4.4%)	66 (6.0%)	37 (3.4%)	194 (17.6%)	**0.004**
	No change	158 (14.3%)	140 (12.7%)	119 (10.8%)	110 (10.0%)	527 (47.8%)	
Downloading any application or started following any social media account concerning Healthy Nutrition	Yes	73 (6.6%)	111 (10.1%)	121 (11.0%)	93 (8.4%)	398 (36.1%)	**0.000**
	No	213 (19.3%)	205 (18.6%)	161 (14.6%)	126 (11.4%)	705 (63.9%)	
Downloading any application or started following any social media account concerning Physical Activity and Exercise	Yes	113 (10.2%)	153 (13.9%)	181 (16.4%)	133 (12.1%)	580 (52.6%)	**0.000**
	No	173 (15.7%)	163 (14.8%)	101 (9.2%)	86 (7.8%)	523 (47.4%)	
During the pandemic, how many hours do you sleep per day?	Less than 7 h	68 (6.2%)	75 (6.8%)	66 (6.0%)	60 (5.4%)	269 (24.4%)	
	7−9 h	172 (15.6%)	179 (16.2%)	149 (13.5%)	136 (12.3%)	636 (57.7%)	**0.012**
	More than 9 h	46 (4.2%)	62 (5.6%)	67 (6.1%)	23 (2.1%)	198 (18.0%)	
Since the beginning of the pandemic, have you noticed a change in your sleep hours per day?	Less	33 (3.0%)	46 (4.2%)	42 (3.8%)	34 (3.1%)	155 (14.1%)	
	More	97 (8.8%)	138 (12.5%)	122 (11.1%)	63 (5.7%)	420 (38.1%)	**0.001**
	No change	156 (14.1%)	132 (12.0%)	118 (10.7%)	122 (11.1%)	528 (47.9%)	
Do you currently smoke (this includes regular cigarettes, electronic cigarettes, hookah, etc.)?	Yes	48 (4.4%)	65 (5.9%)	41 (3.7%)	52 (4.7%)	206 (18.7%)	**0.040**
	No	238 (21.6%)	251 (22.8%)	241 (21.8%)	167 (15.1%)	897 (81.3%)	
Total		286 (25.9%)	316 (28.6%)	282 (25.6%)	219 (19.9%)	1103 (100.0%)	

*Note*: Values are expressed as the number and percentage of total respondents (*n* [%]). Variables are considered significant at *p* < 0.05 and marked in bold.

The significant associations with those who were performing PA are as follows: the male gender (*p* < 0.001), weight loss during the pandemic (*p* < 0.001), decrease or no change in the number of meals during the day (*p* = 0.001), no change in the number of meals during the night (*p* = 0.001), decrease in appetite (*p* = 0.02), downloading any applications or starting to follow any social media accounts concerning healthy lifestyle, nutrition, PA, and exercise (*p* < 0.001). On the other hand, no significant statistical association was observed with each of these parameters: age, social status, number of household members, educational level, occupation, chronic illness, using electronic devices, and smoking status (*p* < 0.05) (Table 2).

### 3.3 Frequency of PA

The male individuals showed that 103 individuals (35.6%) were exercising irregularly, 73 individuals (25.3%) were exercising three to five times a week, and 66 individuals (22.8%) were exercising almost everyday. On the contrary, the female results showed that 358 (44.0%), 112 (13.8%), and 171 (21.0%) were exercising irregularly, three to five times a week, almost everyday, respectively. This concludes that males were exercising regularly from three to five times a week more than females by 1.8‐fold. Also, females were performing exercises at an irregular frequency more than males by 1.2‐fold (Table 3).

Almost everyday PA was noticed among 46.1% of individuals who has been exercising more during the pandemic than before, 21.3% who has no change in their level of exercise during the pandemic, 17.7% who started doing some exercise during the pandemic, and 7.9% who did not start to exercise although they have realized the importance of doing so. This means that those who exercised more during the pandemic than before had regularly exercised everyday more by 5.8‐fold than those who did not start exercise (*p* < 0.001).

Regular exercise is found to be more in the respondents who started smoking before the pandemic more than 10 years (40.0%) than those who started smoking before 2−10 years (19.0%) or those who started smoking the past year (13.3%). As a result, those who were smoking 10 years ago tended to do a regular daily PA more than those who were smoking within the last 10 years and those who started smoking within the last year by 2.1‐fold and 3.1‐fold, respectively (*p* = 0.006) (Table 3).

Moreover, there are significant associations noticed between the respondents who had regular everyday PA and each of these measures: having 4−6 household members (*p* = 0.001), changing weight (*p* = 0.010), no change in the number of daily meals (*p* = 0.028), downloading any applications during the pandemic or starting to follow any social media accounts concerning healthy lifestyle and nutrition (*p* = 0.002) and accounts concerning PA and exercise (*p* < 0.001), no change in utilization of different electronic devices (*p* = 0.037). However, no statistical association is observed with age, social status, educational level, occupation, chronic illness, changes in the number of night meals, appetite, and smoking status (*p* Value for all <0.05) (Table 3).

### 3.4 Changing the level of exercise

**Figure 3:**
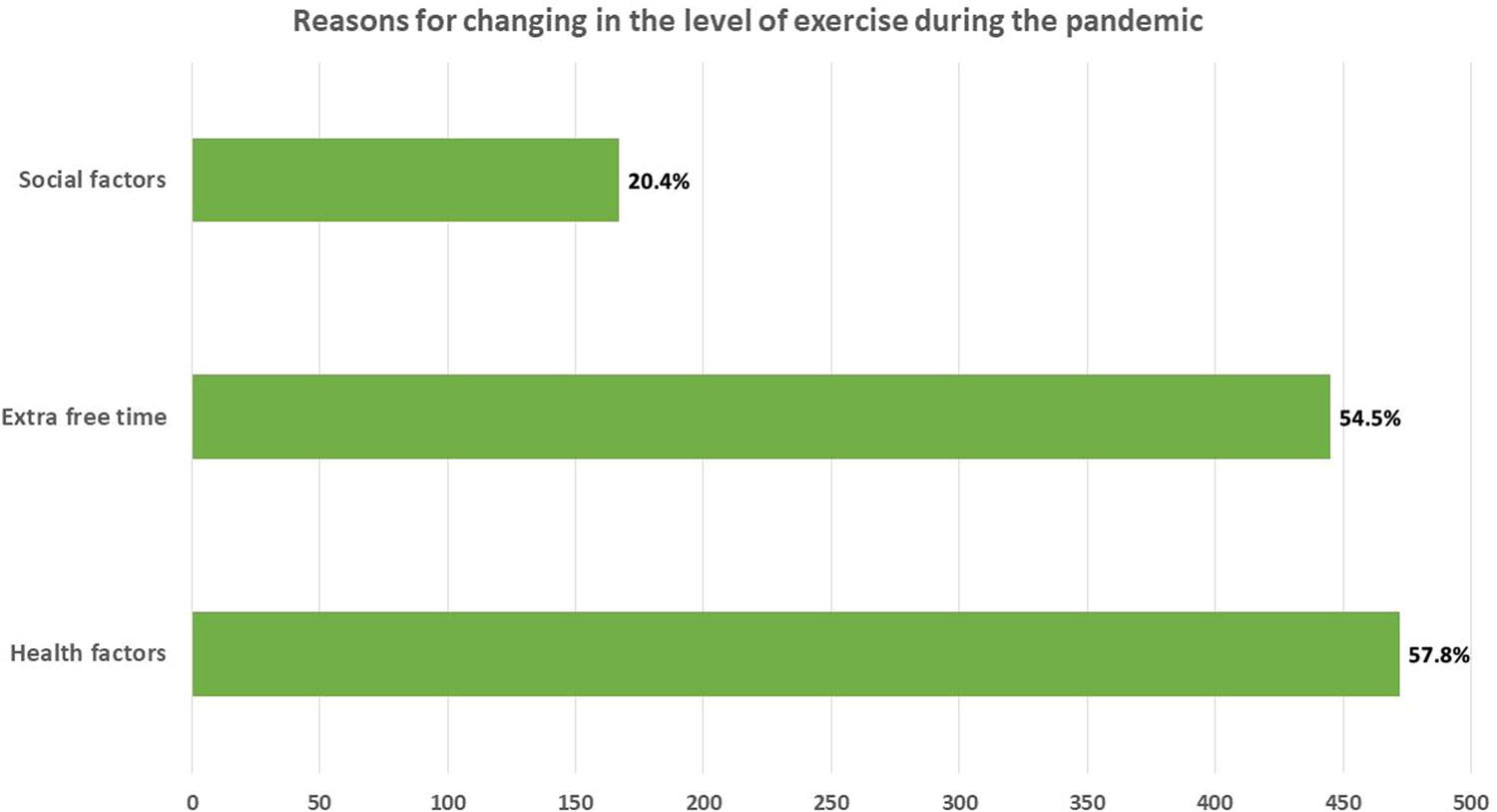
Reasons for changing in the level of exercise during the pandemic or even changing in the way they think of exercise among the respondents who noticed any change in their exercise performance (*n* = 817)

As shown in Figure 3, nearly 25.6% of the participants stated that they have started doing some form of exercise during the pandemic. Those who were children under the age of 18‐year‐old, males, were single, were nonsmokers, and had a diploma or bachelor's degree in a health‐related major were more likely to start doing some form of exercise during the pandemic (*p* < 0.001, 0.001, 0.001, 0.040, 0.002), respectively. What's more, apparently there is a significant association between starting to do some form of exercise only since the beginning of the pandemic and the following parameters: losing weight (*p* < 0.001), decrease in appetite (*p* = 0.004), decrease in the number of daily meals (*p* = 0.003), increase in the number of night meal (*p* = 0.033), increase in sleeping hours per day since the beginning of the pandemic (*p* = 0.001), using less electronic devices during the pandemic (*p* = 0.001), starting to follow social media account concerning healthy nutrition (*p* < 0.001) and PA (*p* < 0.001). Meanwhile, there is no significant association found between occupation, number of household members, and having chronic illnesses with starting to do some form of exercise during the pandemic (*p* value for all >0.05) (Table 4). Almost 219 (19.9%) of the participants who have been regularly exercising before the pandemic was found to be more driven to exercise since the pandemic began. This effect was significantly associated with: age above 35 years, being female, being married, having a Master's degree or PhD, no change in the number of meals during the day or night, no change in weight or appetite, no change in the using of electronic devices during the pandemic, being a smoker, starting to follow social media accounts concerning healthy nutrition and PA (*p* value for all >0.05) (Table 4). In addition, around 57.8% of the respondents said that the main concern about performing PA was due to the health aspects where they realized the importance of exercising in strengthening the immune system against diseases, including COVID‐19. Likewise, 54.5% of the respondents stated that the extra free time due to the lockdown and studying or working from home had significant effects. Only 20.4% of them found that the influence of family members, friends, physicians, or social media affected their level of exercise during the pandemic (Figure 3).

## 4 DISCUSSION

On March 17, 2020, in an attempt to control and limit the spread of COVID‐19, the Jordanian government issued a strict nationwide lockdown that included all sectors sparing essential and health care services. The aforementioned lockdown was tightly monitored by the government and was loosened after 42 days of a curfew. During the lockdown, nearly the entirety of the Jordanian population was homebound and not allowed to travel via automobiles within or intergovernorates. The new global circumstances were sufficient to alter individuals' routines and some lifestyle behaviors including the patterns of physical exercise.^16^ Considering these tremendous changes, this study was designed to better understand Jordanians' physical exercise patterns both before and after the pandemic.

In the current study, the results showed that 43.9% of the respondents reported partaking in various forms of physical exercise. In particular, 21.5% exercised almost everyday, 16.8% exercised three to five times a week, and 41.8% exercised at an irregular frequency. In addition, 28.6% of the study population have realized the importance of exercise but do not necessarily apply it to their daily routine. On the other hand, 25.6% have started doing some form of exercise while 19.9% have regularly exercised before the pandemic and they have been more driven to exercise since the pandemic began, which came in congruence with Ding D et al. who conducted his study across Australia, the UK, and the USA, that the COVID‐19 lockdown may have led to increases in population‐level interest and engagement with PA, which could be potentially explained by attempts to compensate for reduced incidental activities, availability of discretionary time, increased health awareness and recommendations to exercise from the media, governments health authorities (e.g., the WHO).^17^

In a previous study, Ammar et al. found out that there has been a decrease in the number of days/week and min/day of all types of PA during their lockdown by 24% and 33.5%, respectively (*p* < 0.001).^13^ The authors found out that there has been a decrease in the number of days/week and min/day of walking during their lockdown by 35% and 34%, respectively (*p* < 0.001).^13^ Additionally, Gallo LA et al. found that students at Australian University had a significant decrease in the duration of walking and PA in the pandemic (2020) compared to the past 2 years (2018 and 2019) (*p* < 0.05).^18^ Moreover, Seetan et al. reported that 73.1% of the Jordanian medical students had a negative impact of this pandemic on their physical fitness.^19^

Interestingly, respondents who perform PA were significantly associated with the male gender, which agreed with Qin et al. who concluded that amongst 12,107 Chinese adults, the prevalence of insufficient PA among men (55.1%) was significantly lower than among women (59.5%) during their Chinese lockdown (*p* < 0.0001).^20^ On the other hand, this came in disagreement with Rodríguez‐Larrad et al. whose research was conducted through surveys across 13,754 Spanish University students and found out that adaptation to the lockdown, in terms of PA, was handled better by women than by men.^21^ On the other hand, Gallo et al. found that there was no significant difference among males and females regarding PA during the pandemic in 2020 compared with 2018/2019 among Australian University students.^18^ Moreover, respondents who perform PA were significantly associated with those who lost weight during the pandemic, which came in parallelism with Chopra et al. who concluded that among 995 responses collected in India, one‐third of participants gained weight as PA declined during the COVID‐19 pandemic.^22^ Furthermore, according to Galali in a study with a sample size of (*n* = 2137), the frequency of PA was significantly decreased, and 32.4% reported weight gain.^23^

On the other hand, there was no statistical association between respondents who perform PA and social status, the number of household members, educational level, occupation, chronic illness, using electronic devices, smoking status, and age. However, according to Qin in a study with a sample size of (*n* = 12107), Chinese adults aged between 18 and 80, the group with the highest prevalence of insufficient PA was found among young adults aged 20–34 years.^20^ In contrast, a lower prevalence of insufficient PA was found in those aged 55–59 years (41.1%) and over 60 years old (41.3%) during the COVID‐19 lockdown.^20^

There was also a strong association found between the increased number of household members and the frequency of exercise. 81.7% of participants come from households with four or more members, 34% of which exercise in an irregular pattern while 7.8% of participants who are members of households that contain three or fewer members exercised in that same manner. Comparably, the percentage of household members of four or more who exercise almost everyday is 18.6% which exceeds 2.9% representing individuals of household members of three or less in that same category.

In the current study, 31.9% of participants who live in cities were engaging in PA in an irregular manner, compared to 9.6% and 0.3% of participants who live in villages or refugee camps, respectively. It was also found that people living in cities who exercise almost everyday were 15.0% in comparison to 5.9% of participants who live in villages and 0.5% who live in refugee camps. Similar percentages were also found regarding exercising once or twice a week and three to five times a week.

In an attempt to understand the general trends affecting PA during the COVID‐19 pandemic, an online survey was conducted by Lesser in Canada and concluded that 40.5% of inactive individuals were more likely to become less active during the pandemic while a smaller percentage of already active individuals 22.4% showed increased PA.^24^ When assessing the same concept amongst our Jordanian sample, participants were asked about noticing any change in their level of exercise or even a change in the way they perceive exercise since the beginning of the pandemic was found as follow, 25.9% of participants reported no change, and 13.1% of them reported exercising irregularly. 28.6% of people reported realizing the importance of exercise without necessarily applying it to their daily routine. While 9.2% of people who exercised before the pandemic but were more driven to exercise since it began reported they exercised almost daily compared to 2.0% of participants in the same category who reported irregular exercise.

To understand the association between the COVID‐19 pandemic and the use of social media, Ali et al. and colleagues found that examined 251 Pakistani adolescents, that there was a significant difference in the median difference of time spent on social media before the outbreak (3.0 ± 32.46) and time spent on social media after the outbreak (6.0 ± 3.52) in a single day.^25^ The previous findings of Ali et al. are compatible with the finds of Gallè et al. who concluded a general trend of increase in the use of electronic devices amongst 1430 Italian undergraduate students by (+52.4 min/day).^9^ Similarly, our research reflected a significant association between the frequency of exercise and downloading or started following social media accounts concerning PA and exercise in specific. This could be in fact due to the increase in electronic devices and social media use in general.

The current findings showed that respondents who are nonsmokers were more likely to start doing some sort of exercise during the pandemic compared to smokers (which includes regular smoking, electronic smoking, hookah, etc.). In addition, children less than 18 years old were more likely to have started exercising during the pandemic. While 16.8% of individuals, who are between 18 and 35 years, reported no change in the level or way at which they exercised since the beginning of the pandemic. Moreover, we found that 19.9% of respondents from the previous category were more likely to realize the importance of exercise but not necessarily apply it to their daily routine, while 18.2% reported starting to perform some form of exercise since the start of the pandemic, hence age has been closely associated with the level of exercise. On the other hand, a Belgian study conducted by Constandt et al. found that participants of (the 18–34) and (35–54) age groups are more likely to increase exercise during the lockdown.^26^ The same study showed that people with higher educational levels have a greater chance to exercise more during the pandemic than those with lower educational levels.^26^ However, people who have diplomas or bachelor's degrees in health‐related majors were more likely to start exercising during the pandemic. This may be justified by the results of Droomers et al., who concluded that there was a statistically significant association between the level of education and the tendency to perform a PA and such that the probability to decrease PA was linked to individuals with lower levels of education.^27^

Both gender and social status were also linked to the level of physical exercise. In particular, 21.8% of females reported realizing the importance of exercise without necessarily applying it to their routine followed by 20.5% of females who have started to do some form of exercise since the start of the pandemic. All while 7.3% of males reported no change in their level of exercise. As for the social status, we found that single people were more likely to exercise before and after the start of the pandemic.

## 5 CONCLUSION

In conclusion, our study showed that COVID‐19 and its lockdown had a significant impact on the population's lifestyles including PA. Most of our sample, who were doing any form of physical exercise during the COVID‐19 pandemic, experienced a change in their level of exercise either increased, decreased, or started doing PA since the beginning of the pandemic. These changes were mostly attributed to realizing the importance of exercising in strengthening the immune system against diseases, extra free time, and social factors. In general, the study showed that respondents who were doing any form of exercise during the pandemic were more likely to adopt a healthy lifestyle during the pandemic in comparison with nonperforming‐exercise respondents. Moreover, among performing‐exercise respondents themselves, those who increased their activity during the pandemic were also more likely to adopt a healthy lifestyle during the pandemic.

## 6 LIMITATIONS

As previously mentioned in the methodology section, a multisection online questionnaire was designed and distributed in November. We faced a potential limitation in recall bias since the Jordanian government‐enforced quarantine in March. There could also be potential reporting bias as the questionnaire was self‐reported. In addition, another limitation could be a small ratio of male to female participants, which was 1:3.4. Taking into consideration all the predicted limitations, we managed to subdue the aforementioned by collecting a sufficient and representative sample and using appropriate statistical tools.

## AUTHOR CONTRIBUTIONS

*Conceptualization*: Mazhar S. Al Zoubi and RMai Alzoubi. *Study design, data collection, and data analysis*: Sajeda Awadi, Almu'atasim Khamees. *Writing‐Original draft preparation*: Almu'atasim Khamees, Sajeda Awadi, Walaa Almdallal, Mai Alzoubi, Lana Talafha, Ahmad M. Alsheikh and Baha Aldin Faiyoumi. *Writing‐reviewing and editing the final manuscript*: Raed M. Al‐Zoubi, and Mazhar S. Al Zoubi. All authors reviewed, contributed, and approved the final manuscript version.

## CONFLICT OF INTEREST

The authors declare no conflict of interest.

## TRANSPARENCY STATEMENT

The lead author Raed M. Al‐Zoubi affirms that this manuscript is an honest, accurate, and transparent account of the study being reported; that no important aspects of the study have been omitted; and that any discrepancies from the study as planned (and, if relevant, registered) have been explained.

## Data Availability

The supporting data of the findings of this study are available on request from the corresponding author.

## APPENDIX A:

### A:.1

**Health practices related to the covid‐19 pandemic**

We appreciate your participation in this study!

The researchers aim to detect the relationship between the Covid‐19 pandemic and certain health and lifestyle choices of the Jordanian population.

All the questions in this survey are multiple choice questions. Please choose the answer that suits you best.

**Section 1 of 6: Demographics**

**Q: Age**:

− Under 18 years
− 18−25 years
− 26−35 years
− 36−50 years
− 51−65 years
− Over 65 years

**Q: Gender**:

− Male
− Female

**Q: Social status**:

− Single
− Married

**Q: If your social status is married, do you have any children?**

− Yes
− No

**Q: Number of household members**

− 3 or less
− 4−6
− 7 or more

**Q: Educational level**:

− High school diploma or still in school.
− Diploma or bachelor's degree in a health‐related major (Nutrition, Medicine, Dentistry, Allied health sciences).
− Diploma or bachelor's degree in non‐health‐related major.
− Master's degree or PhD.

**Q: Occupation**:

− I work in a health‐related field.
− I work in a non‐health‐related field.
− I don't work (student, retired, housewife, etc.).

**Q: Place of residence**

− City
− Village
− Refugee camp

**Q: Governorate**

− Amman
− Irbid
− Al‐Zarqa
− Al‐Karak
− Al‐Balqa
− Aqaba
− Ma'an
− Al‐Mafraq
− Ajloun
− Madaba
− Jerash
− Al‐Tafila

**Q: Do you suffer from any chronic illnesses?**

− Yes
− No

**Q: Do you currently perform any form of physical exercise (this includes walking, jogging or any activity requiring physical exertion)?**

− Yes
− No

**Section 2 of 6: Physical activity**

**Q: Frequency of exercise**

− Irregularly.
− Once or twice a week.
− Three to five times a week.
− Almost everyday.

**Q: Have you noticed any change in your level of exercise or even a change in the way you think of exercise since the beginning of the pandemic?**

I have not noticed any change.

I have realized the importance of exercise but do not necessarily apply it to my daily routine.

I have started doing some form of exercise.

I regularly worked out before the pandemic but I am even more driven to exercise since the pandemic began.

**Q: If you have noticed a change regarding your exercise level, what do you feel is the reason behind this change?**

Extra free time due to lockdown and studying/working from home.

The health aspect (I realized the importance of exercising in strengthening the immune system against diseases including Covid‐19).

The social aspect (the influence of family members, friends, physicians or social media).

**Section 3 of 6: Eating habits**

In this section, we want to know about your eating habits and diet and what changes you might have made in the current circumstances.

*Please answer these questions regarding your eating habits (before the start of the pandemic)*.

Eating habits (before the start of the pandemic).

**Q: Fast food intake**:

− Once a month or less.
− Once a week.
− Three to four times a week.
− Almost daily.

**Q: Sugar intake (this includes sweets and desserts, sodas, sweetened juices, biscuits, chocolate, etc.)**:

− Once a month or less.
− Once a week.
− Three to four times a week.
− Almost daily.

**Q: Fat and oil intake (this includes any foods that contain vegetable oil or any hydrogenated or trans‐fats)**:

− Once a month or less.
− Once a week.
− Three to four times a week.
− Almost daily.

**Q: Fresh fruit and vegetable intake**:

− Once a month or less.
− Once a week.
− Three to four times a week.
− Almost daily.

*Please answer these questions regarding your eating habits (after the start of the pandemic)*.

Eating habits (after the start of the pandemic).

**Q: Fast food intake**:

− Once a month or less.
− Once a week.
− Three to four times a week.
− Almost daily.

**Q: Sugar intake (this includes sweets and desserts, sodas, sweetened juices, biscuits, chocolate, etc.)**:

− Once a month or less.
− Once a week.
− Three to four times a week.
− Almost daily.

**Q: Fat and oil intake (this includes any foods that contain vegetable oil or any hydrogenated or trans‐fats)**:

− Once a month or less.
− Once a week.
− Three to four times a week.
− Almost daily.

**Q: Fresh fruit and vegetable intake**:

− Once a month or less.
− Once a week.
− Three to four times a week.
− Almost daily.

**Q: (Since the start of the pandemic) have you added any of these ingredients (garlic, onion, and ginger) to your diet, or increased your intake of them?**

− Yes
− No

**Q: (Since the start of the pandemic) have you noticed any change in your weight?**

− I have gained weight.
− I have lost weight.
− My weight hasn't changed.
− I don't know.

**Q: (Since the start of the pandemic) has the number of meals you eat in a day changed?**

− The number of meals I have in a day has increased.
− The number of meals I have in a day has decreased.
− The number of meals I have in a day hasn't changed.

**Q: (Since the start of the pandemic) has the number of meals you eat during nighttime increased?**

− Yes
− No

**Q: (Since the start of the pandemic) have you noticed any change in your appetite?**

− My appetite has increased.
− My appetite has decreased.
− My appetite has not changed.
− There have been a lot of changes to our society due to the COVID‐19 pandemic. The researchers are trying to understand the effect of these changes on people's eating practices.

**Q: Please select from the following statements what you think has affected your eating habits and diet during the pandemic**:

− The economic aspect (being off work or losing a job, limited availability of food products and produce, and the closing of restaurants and cafes).
− Extra free time due to lockdown and studying/working from home.
− The health aspect (realizing the importance of a healthy and balanced diet in strengthening the immune system against diseases including COVID‐19).
− The social aspect (the influence of family members, friends, physicians or social media).

**Section 4 of 6: Social Media and Sleep**

**Q: During the pandemic, did you download any application or started following any social media account concerning any of the following**:

Healthy Nutrition

− Yes
− No

Physical Activity and Exercise

− Yes
− No

Medical Applications/accounts/websites

− Yes
− No

**Q: During the pandemic, did you notice a change related to your utilization of different electronic devices (e.g. mobile phones, laptops, television, etc…)?**

− I started using electronic devices more.
− I started using electronic devices less.
− I did not notice any change.

**Q: If your answer to the previous question was (I started using electronic devices more), what do you think caused that?**

*You can choose more than one option.

Working online.

Leisure and spare time.

Studying online.

**Q: Before the pandemic, how many hours did you sleep per day?**

− Less than 7 h
− 7−9 h
− More than 9 h

**Q: During the pandemic, how many hours do you sleep per day?**

− Less than 7 h
− 7−9 h
− More than 9 h

**Q: Since the beginning of the pandemic, have you noticed a change with regard to your sleep hours per day?**

− I started sleeping less.
− I started sleeping more.
− I have not noticed any change concerning my sleep hours.

**Q: Do you currently smoke (this includes regular cigarettes, electronic cigarettes, hookah, etc.)?**

− Yes
− No

**Section 5 of 6: Smoking**

**Q: When did you start smoking?**

− During the past year.
− 2−10 years ago.
− More than 10 years ago.

**Q: Since the beginning of the pandemic, have you noticed any change related to your smoking habits?**

− I started smoking more.
− I started/tried smoking less or tried to quit.
− I have not noticed a change.

**Q: If you have noticed a change in your smoking habits (whether an increase or a decrease in the frequency of smoking), what do you think caused that change?**

*You can choose more than one answer.

Days off from work, losing a job, income limitations.

The general lockdown of cafés and public smoking lounges, and so forth.

Health factor (I realized the adverse effects smoking might have on the immune system that is vital to fight off diseases like COVID‐19).

Social factor (influence of family members and friends).

Psychological factor (different stressors due to the financial burden of losing a job or online working or studying, etc…).

Free time and boredom due to the general lockdown and quarantine.

**Section 6 of 6: Nutritional supplements (vitamins and minerals like iron and zinc)**

**Q: Before the pandemic, were you interested in consuming nutritional supplements like vitamins and minerals including iron and zinc?**

− Yes
− No

**Q: Since the beginning of the pandemic, did you introduce nutritional supplements to your diet or increase the amount that you consume?**

− Yes
− No

**Q: Do you think nutritional supplements play a role in boosting the immunity to fight off diseases like COVID‐19?**

Yes, I think nutritional supplements can boost immunity and protect against the disease.

I do not think nutritional supplements have any role in that.

I do not know.

*Thank you for participating in our questionnaire. We wish you, your family and your loved one's health and well‐being.
